# Supplementary material for: Increased Enzymatic Activity of Acetylcholinesterase Indicates the Severity of the Sterile Inflammation and Predicts Patient Outcome following Traumatic Injury
Source: Biomolecules. 2023 Jan 31;13(2):267. doi: 10.3390/biom13020267 (PMC9952955; doi:10.3390/biom13020267)

measurement	**volunteers**	**trauma patients**	
		time point of the measurement	
		hospital admission	stabilization (4-12 hours following admission)
AChE (U/g Hb)	37 (32-41)	41 (36-45)	41 (36-46)
CRP (mg/L)		0 (0-2)	22 (6-44)
WBCC (nL^{-1})		13 (9-17)	10 (9-13)

Supplementary Table S1: Measurements obtained from volunteers and injured patients. Data are presented as medians with interquartile range. AChE – acetylcholinesterase; CRP – C-reactive protein; WBCC – white blood cell count.

Supplementary Figure S1

Strong correlation between the disease severity scores and the length of ICU stay

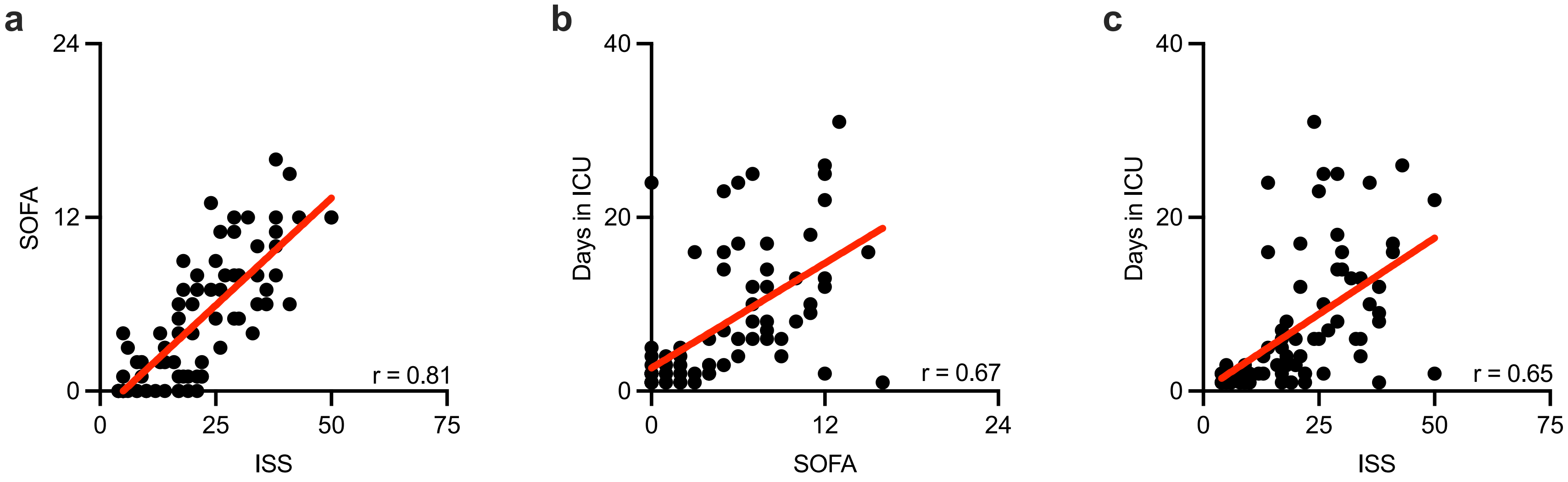

Supplement: Supplementary file 1 [file biomolecules-13-00267-s001.zip › biomolecules-2152554-supplementary.pdf]
